# Beneficial short-term effect of autogenic drainage on peripheral resistance in childhood cystic fibrosis disease

**DOI:** 10.1186/s12890-022-02039-2

**Published:** 2022-06-21

**Authors:** Plamen Bokov, Michèle Gerardin, Géraldine Brialix, Emmanuelle Da Costa Noble, Romain Juif, Antonia Vital Foucher, Laurence Le Clainche, Véronique Houdouin, Benjamin Mauroy, Christophe Delclaux

**Affiliations:** 1 Service de Physiologie Pédiatrique-Centre du Sommeil, INSERM NeuroDiderot, Université de Paris, Hôpital Robert Debré, AP-HP, 48, boulevard Sérurier, 75019 Paris, France; 2 Service de Pneumopédiatrie, Centre de Ressources et de Compétences de la Mucoviscidose, Hôpital Robert Debré, AP-HP, 75019 Paris, France; 3 Laboratoire JA Dieudonné, CNRS, Université Nice Côte d’Azur, 06108 Nice, France

**Keywords:** Chest physiotherapy, Impulse oscillometry, Lung model, Cystic fibrosis, Childhood

## Abstract

**Background:**

Airway clearance techniques are supposed to be a necessary adjunct for the enhancement of impaired peripheral clearance in cystic fibrosis (CF). The objective was to assess the effect of one physiotherapy session (autogenic drainage: AD) on mucus clearance (sputum wet weight) and impulse oscillometry system (IOS) indices, including those obtained from extended Resistance-Inertance-Compliance (eRIC) modelling, considering the degree of bronchial congestion.

**Methods:**

Thirty children with CF (median age: 12.7 years) in a stable condition prospectively underwent IOS measurements at baseline and after AD. They were divided in two groups: with (visual analog scale of bronchial congestion by the physiotherapist ≥ 5/10) and without (scale < 5/10) bronchial congestion. Paired-comparison of the effects of AD on airway resistance measurements was done with Wilcoxon test.

**Results:**

The congestion scale correlated with the wet weight of sputum production during the session (Pearson test: *p* < 0.0001, R = 0.66). Ten children had bronchial congestion and 20 were without congestion. In the whole group, R5–20 Hz significantly decreased after AD (*P* = 0.049), which was related to a decrease in the children with congestion (*P* = 0.025), whereas it was not significantly modified in the children without congestion (*P* = 0.327). The eRIC model allowed the calculation of the peripheral resistance of the respiratory system, which also decreased in the children with congestion (*P* = 0.037), however, not modified in the children without congestion (*P* = 0.390).

**Conclusion:**

One session of autogenic drainage has the ability to decrease peripheral resistance obtained from IOS measurements, more specifically in children with CF with moderate to severe bronchial congestion.

***Trial registration*:**

ClinicalTrials.gov Identifier: NCT04094441.

## Background

In 1985, Kirilloff and colleagues posed the question, “Does chest physical therapy work?”, and in their review, they concluded that chest physical therapy has frequently been shown to be of benefit in cystic fibrosis (CF) [1]. Generally, patients with other chronic lung diseases have shown improvement following chest physical therapy if they produce large volumes of sputum [1]. Moreover, Bateman and colleagues reported that radioaerosol clearance increased fivefold in the central and intermediate lung regions and fourfold in peripheral lung regions when compared with control days [2], suggesting the ability of airway clearance techniques to mobilise distal airway secretions. These findings confirmed that cough only partially compensated for impaired mucociliary clearance and airway clearance techniques were a necessary adjunct for the enhancement of impaired peripheral lung clearance [2].

Airway clearance techniques are usually commenced as soon as the diagnosis of CF is made. In a systematic review, Warnock and Gates evaluated the different modalities of airway clearance techniques in CF [3]. The results of this review showed that airway clearance techniques had short-term effects in the terms of increasing mucus transport [3]. Overall, these techniques seem to provide similar effects in terms of airway clearance and recent guidelines state that, in most cases, there is little evidence to support the use of one technique over another [4, 5]. In a long-term comparative study in adolescents with CF, autogenic drainage (AD) was as effective as postural drainage, and participants showed strong preference for AD [6]. The effects of airway clearance techniques on pulmonary function indices remain debated, since either no effect, detrimental or beneficial effects have been shown, mostly using spirometry [3].

Based on this background, we hypothesised that AD in children with CF could improve peripheral airway indices, depending on the volume of sputum cleared during the session. Sakarya and colleagues showed that the indices obtained from the impulse oscillometry system (IOS) were improved after treatment of an acute exacerbation in children with CF [7]. IOS is a variant of the forced oscillation technique, which allows passive measurement of total respiratory system impedance. In this method, indices describing resistance to airflow (respiratory system resistance, RRS) and reactive indices that mostly relate to the efficient storage and return of energy by the lung (respiratory system reactance, XRS) are obtained [8]. Since IOS measurements involve frequencies and impedances, it is possible to correlate the measurements to respiratory system models consisting of analogous electrical components [8], to provide parameter estimates that are physiologically more realistic and relevant. The extended Resistance-Inertance-Compliance (eRIC) model gives access to two peripheral markers of small airway disease, peripheral compliance and resistance of the respiratory system [8]. The objective of this prospective study in children with CF was to assess the effect of a single AD session on mucus clearance (sputum wet weight) and IOS indices, including those obtained from eRIC modelling, taking into account the degree of bronchial congestion.

## Methods

### Design (cross-sectional study)

This interventional study was registered (ClinicalTrials.gov Identifier: NCT04094441) on September 19, 2019.

**Fig. 1:**
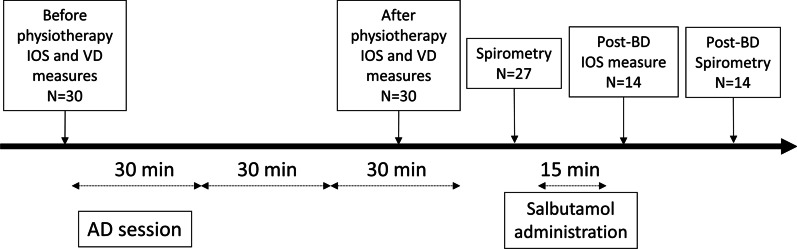
Timeline of study procedures. IOS denotes impulse oscillometry system; VD denotes anatomical dead space; AD denotes autogenic drainage. 27/30 children performed spirometry after the AD session and among them 14 had a bronchodilator test (salbutamol 400 µg) due to airflow limitation

The subjects did not perform physical therapy on the morning of the tests and received no bronchodilator or mucolytic treatment in the 12 h preceding the tests. Since forced expiration has been associated with cough, spirometry was not performed at baseline in order not to induce a bias related to expectoration of mucus. The timeline of study procedures is provided in Fig. 1.

### Patients

Children 4–18 years with CF followed at the Cystic Fibrosis Centre (CRCM) at Robert Debré hospital were enrolled during their annual check-ups. All subjects were clinically stable (no antibiotics for an exacerbation in the preceding 4 weeks). This study conformed to the standards set by the latest revision of the Declaration of Helsinki and was approved by an ethics committee (CPP SUD-OUEST et OUTRE MER II, n° IDRCB: 2017-AO2426-47). The parents of the children gave written informed consent; assent was also obtained from children.

### Chest physiotherapy

Assessment of AD session: before the session, the degree of bronchial congestion was evaluated by both the child (absence versus presence) and physiotherapist (based on auscultation, visual analogic scale, from 0 [absence] to 10 [maximum]). The children with CF were then divided in two groups according to the visual analogue scale: < 5/10 (absence to mild bronchial congestion denoted no congestion) and ≥ 5/10 (moderate to severe bronchial congestion denoted congestion). All sputum samples produced during the session were collected and weighed (within 15 min after the end of the session). The sputum weight was determined in the pulmonary function test unit by an independent investigator, who was blinded to the congestion rating.

AD was performed by four experienced chest physiotherapists devoted only to children with CF. AD is an airway clearance technique developed by Chevallier that is based on non-forced expiration during controlled breathing at different levels of vital capacity [9, 10]. Briefly, while sitting, the patient first performs diaphragmatic breathing at low lung volume following a cycle of slow inspiration through the nose to total pulmonary capacity, followed by a pause of three seconds, then non-forced exhalation through the nose or mouth to residual volume. These steps are repeated until mucus movement can be felt. The entire breathing cycle is repeated at progressively increasing lung volumes, so that mucus is moved from the small to the mid-sized to the large airways. Finally, mucus is evacuated from the trachea by huffing. In this study, the cycles were repeated for a total AD session of approximately 30 min.

Children practiced chest physiotherapy on a usual basis (three times a week with a physiotherapist).

### Pulmonary function tests

Impedance of the respiratory system was measured using an IOS (Master Scope Body, Carefusion Technologies, Yorba Linda, California, USA), as previously described [8, 11]. The quality criterion for IOS measurements was a coherence value ≥ 0.6 at 5 Hz [11]. Five trials of IOS were repeated and we kept only the three recordings yielding the lowest coefficient of variation of impedance at 5 Hz for analysis, as previously described [11] and accordingly to recent guidelines [12]. We used the following IOS variables: impedance at 5, 10, 15, 20, 25, 30 and 35 Hz, resistance and reactance at 5 Hz and 20 Hz, fall in resistance between R5 and R20 (R5–20 Hz), area under the reactance curve (AX) and resonance frequency (Fres). The % predicted and z-scores of the IOS variables were calculated according to Gochicoa-Rangel et al. [13]. The assessment of IOS was between 30 and 60 min after AD session based on the results of Hortal and Hjelte [14].

We used one mechanistic model capable of accounting for significant frequency dependence of the respiratory impedance, which has previously been described [8]. The RIC model is constituted by a Resistance, an Inertance (IRS) and a Compliance of the respiratory system. In the eRIC model, resistance is partitioned in the central resistance (R_central_RS) and peripheral resistance (R_peripheral_RS) of the respiratory system, while the peripheral compliance of the respiratory system (CpRS) includes the parenchymal and chest wall compliances. The model was fitted to the impedance data (5–35 Hz) and the minimization of a performance index allowed the calculation of model indices [8]. To determine the relative appropriateness of the various inverse model topologies, we used the corrected Akaike information criterion (AICc) [8].

Anatomical dead space (VD) measurement was obtained using the apparatus for multiple breath washout measurement (Exhalyzer D, Eco Medics AG, Duernten, Switzerland with Spiroware 3.1.6 software) based on the analysis of expired CO_2_ pressure. The mean VD obtained from five respiratory cycles was recorded. VD (cm^3^) was further normalised by height (cm), due to the close relationship between these two factors. One may hypothesise that mucus clearance due to AD could increase “free” anatomical dead space.

Spirometry (Master Scope Body, Carefusion Technologies, Yorba Linda, California, USA) was obtained according to international guidelines [15] in older (aged ≥ 6 years) children only. The z-scores of the spirometry indices were calculated based on Global Lung Initiative-2012 reference values [16]. A bronchodilator test (salbutamol 400 µg using an inhaler device) was further performed in children with airflow limitation (z-score of FEV_1_/FVC <  − 1.645). A secondary objective was to evaluate the peripheral effects of salbutamol.

### Statistical analyses

The power calculation for this study was based on data published for CF patients in a study that evaluated the changes in respiratory system resistance and reactance in adult CF subjects after a single AD session [17]. As a result, we planned to include a similar number of patients (n = 30). Our primary objective was to show that peripheral resistance (R5–20 Hz) decreased after AD. Using 30 participants, it allowed to show a decrease in R5–20 Hz of 0.05 kPa/L/s after one AD session with a Standard Deviation of 0.095 for its change (α, two-tailed, of 0.05 and β of 0.20) using the T statistic (with a non-centrality parameter) [18].

The results are expressed as median [25th–75th percentile] for continuous data and as frequency (percentage) for categorical data, except when stated. Comparisons between baseline and after AD or between after AD and after salbutamol were performed using the Wilcoxon rank paired test. Comparisons of the continuous variables between the two groups of children were performed using the Mann–Whitney U test [19]. Categorical variables were compared using a chi-square test with Fisher correction when needed. The other tests are described in the text. A *P* value < 0.05 was deemed significant. No correction for multiple testing was done due to the pathophysiological design of the study [20]. Statistical analyses were performed with StatView 5.0 (SAS institute, Cary, North Carolina, USA) software.

## Results

### IOS and eRIC model qualities

The mean (± standard deviation) of the coefficients of variation of Fres, R20Hz, R5Hz, R5–20 Hz, X5Hz and AX were 6 ± 3, 5 ± 4, 6 ± 3, 7 ± 4, 10 ± 10, 12 ± 11; respectively.

The intraclass correlation coefficient within-test of Fres, R20Hz, R5Hz, R5–20 Hz, X5Hz and AX were excellent: 0.95 [0.92; 0.98], 0.98 [0.96; 0.99], 0.98 [0.95; 0.99], 0.98 [0.95; 0.99], 0.94 [0.91; 0.97], 0.93 [0.91; 0.97]; respectively.

**Fig. 2:**
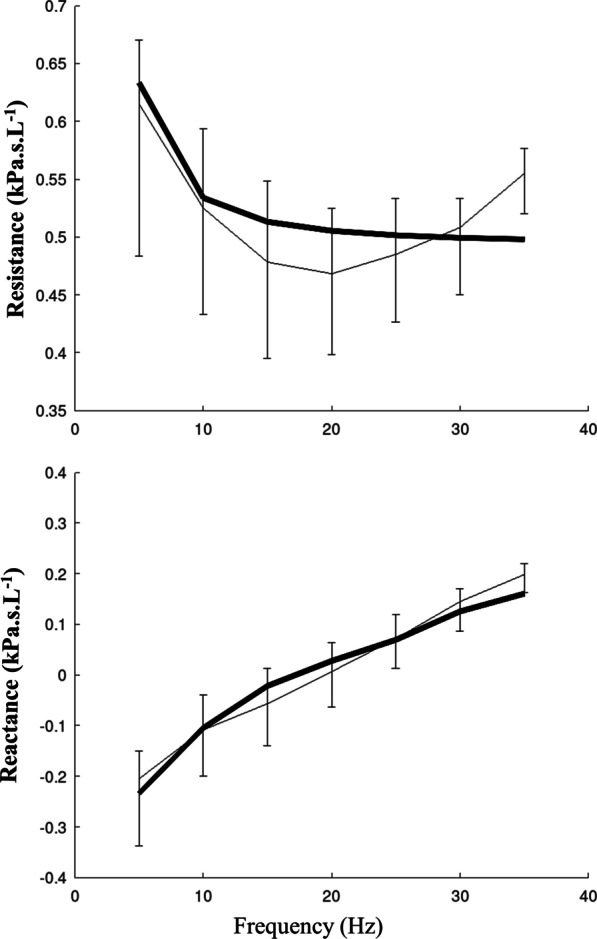
The impedance spectra obtained by fitting the model. Respiratory system resistance (*upper panel*) and reactance (*lower panel*) obtained by fitting the model (thick solid lines) to the baseline data of the experimental measurements represented by the median values and the 25th and 75th percentiles for each frequency (thin lines). Overall, the model adequately fitted experimental data

Performance index at baseline, after physiotherapy and after salbutamol were 0.0095 [0.0048; 0.0198], 0.0068 [0.0046; 0.0093] and 0.0073 [0.0045; 0.0138] kPa^2^ s^2^ L^−2^, respectively. The corrected Akaike information criterion (appropriateness of the various inverse model topologies) at baseline, after AD and after salbutamol were − 86 [− 96; − 76], − 91 [− 96; − 86] and − 90 [− 97; − 81], respectively. Overall, the model adequately fitted the experimental data (Fig. 2).

### Between-group comparisons

The clinical and functional characteristics of the 30 enrolled children with CF are described in Tables 1 and 2, respectively. The range of age was 5 to 18 years, 4 children were younger than 7 years and 6 children were older than 16 years. The congestion score given by the physiotherapist before the session correlated with the wet weight of sputum production during the session (Pearson test, *P* < 0.0001, R = 0.66). There was moderate agreement between the child and the physiotherapist (% of agreement: 73%, Cohen’s kappa: 0.478) (Table 1). Results of the pulmonary function tests were not significantly different between the two groups of children (Table 2).

**Table 1:** Clinical characteristics of the 30 CF children

Characteristics	With bronchial congestion N = 10	Without bronchial congestion N = 20	Between 2 groups (with and without) at baseline
Ethnicity C/B/A/M	9/0/1/0	16/2/0/2	0.253
Age, years	14.6 [9.7; 16.3]	11.8 [7.9; 14.5]	0.333
Sex, female/male	7/3	5/15	**0.045**
Weight, kg	40.5 [33.8; 48.0]	40.0 [23.0; 50.5]	0.552
Weight z-score	− 0.60 [− 0.84; − 0.03]	− 0.66 [− 1.13; 0.04]	0.391
Height, cm	150 [139; 160]	146 [122; 160]	0.843
Height z-score	− 0.44 [− 1.03; 0.16]	− 0.75 [− 1.04; 0.00]	0.644
BMI z-score	− 0.48 [− 0.58; − 0.03]	− 0.34 [− 0.89; 0.71]	0.509
Pancreatic insufficiency, n	10	17	0.519
Previous pseudomonas aeruginosa colonisation, n	10	13	0.093
Dornase alfa/azithromycin, n	9/3	17/5	ND
Inhaled β-agonist/corticosteroid, n	1/1	3/3	ND
CFTR modulator therapy, n	4	5	ND
*Mutation*			
Homozygous /heterozygous/other*	4/4/2	7/6/7	0.689
Score (VAS) of congestion	6.5 [5.0; 7.0]	2.0 [1.0; 3.0]	ND
Sputum weight, g	7.9 [6.1; 12.1]	1.7 [0.9; 4.5]	** < 0.001**
Congestion absent/present^#^	1/9	13/7	**0.007**

Ethnicity Caucasian/Black/Asian/Mixed; BMI denotes Body Mass Index; VAS denotes Visual Analogical Score

^*^ homozygous and heterozygous ΔF508 mutations

^#^based on child’s opinion, absent = absent (or almost absent), present = frank presence

VAS denotes visual analog scale and ND denotes not done. Bold *P* values are those < 0.05

**Table 2:** Functional characteristics of the 30 CF children

Characteristics	With bronchial congestion			Without bronchial congestion			After versus before AD, all patients	After salbutamol versus before all patients	Between 2 groups (with and without) at baseline
	N = 10			N = 20					
Lung function	Before AD	After AD	After salbutamol	Before AD	After AD	After salbutamol			
VD, cm^3^	80 [71; 112]	90 [75; 104]	ND	76 [44; 121]	85 [55; 120]	ND	0.097		0.509
VD/height, cm^2^	0.53 [0.46; 0.70]	0.61 [0.55; 0.66]	ND	0.53 [0.37; 0.70]	0.60 [0.41; 0.75]	ND	0.098		0.660
*Spirometry, patients, n*		10	7		17	7			
FEV_1_, L	ND	1.66 [1.14; 2.32]	1.87 [1.38; 2.42]	ND	2.01 [1.56; 2.59]	1.81 [0.97; 2.49]		**0.007**	
FEV_1_, z-score	ND	− 2.49 [− 3.51; − 2.09]	− 2.30 [− 3.47; − 1.80]	ND	− 0.72 [− 2.41; 0.14]^$^	− 2.38 [− 3.89; − 2.25]			
FVC, L	ND	2.33 [1.55; 3.31]	2.38 [1.89; 3.35]	ND	2.73 [1.97; 3.54]	2.51 [1.77; 3.47]		0.195	
FVC, z-score	ND	− 1.53 [− 2.66; − 0.35]	− 1.18 [− 2.34; − 1.11]	ND	− 0.16 [− 1.52; 0.48]	− 1.54 [− 2.03; − 0.35]			
FEV_1_/FVC	ND	0.75 [0.70; 0.77]	0.78 [0.72; 0.79]	ND	0.81 [0.70; 0.83]	0.69 [0.59; 0.76]		0.064	
FEV_1_/FVC, z-score	ND	− 2.08 [− 2.53; − 1.44]	− 1.84 [− 2.31; − 1.68]	ND	− 1.04 [− 2.31; − 0.84]	− 2.39 [− 3.48; − 1.49]			
*IOS, patients, n*	10	10	10	20	20	20			
Fres, Hz	20.97 [14.34; 23.93]	18.82 [13.88; 26.12]	11.9 [11.2; 25.3]	19.21 [13.76; 25.13]	18.99 [14.74; 22.95]	18.3 [14.5; 25.5]	0.943	**0.016**	
Fres, z-score	0.85 [− 0.70; 1.02]	0.54 [− 0.87; 2.61]	− 0.90 [− 3.79; 2.35]	0.46 [− 1.01; 1.89]	0.18 [− 0.87; 0.96]	0.17 [− 0.84; 1.12]			0.481
R20Hz, kPa/L/s	0.47 [0.31; 0.58]	0.43 [0.28; 0.54]	0.43 [0.29; 0.52]	0.47 [0.39; 0.61]	0.49 [0.39; 0.61]	0.43 [0.33; 0.65]	0.607	0.075	
R20Hz, z-score	1.53 [0.84; 2.25]	1.60 [0.70; 2.18]	1.59 [0.32; 2.00]	1.64 [1.16; 2.68]	1.70 [1.04; 2.42]	1.62 [1.31; 2.37]			0.660
R5Hz, kPa/L/s	0.64 [0.38; 0.80]	0.60 [0.37; 0.75]	0.41 [0.35; 0.76]	0.59 [0.45; 0.88]	0.61 [0.46; 0.85]	0.51 [0.42; 1.16]	0.217	**0.034**	
R5Hz, z-score	1.31 [0.51; 2.35]	1.10 [0.20; 2.56]	0.84 [0.15; 1.69]	1.32 [0.54; 2.46]	1.17 [0.54; 1.85]	1.17 [0.83; 3.77]			0.895
R5–20 Hz, kPa/L/s	0.17 [0.06; 0.22]	0.12 [0.05; 0.22]	0.07 [0.02; 0.23]	0.15 [0.07; 0.32]	0.14 [0.06; 0.19]	0.11 [0.07; 0.34]	**0.049**	0.278	
R5–20 Hz, z-score	0.47 [0.04; 1.70]	0.33 [− 0.32; 1.42]	0.25 [− 0.36; 1.12]	0.55 [− 0.42; 1.57]	0.49 [− 0.49; 0.87]	0.62 [0.30; 2.00]			0.509
X5Hz, kPa/L/s	− 0.24 [− 0.34; − 0.20]	− 0.23 [− 0.33; − 0.19]	− 0.16 [− 0.33; − 0.14]	− 0.19 [− 0.31; − 0.15]	− 0.20 [− 0.27; − 0.15]	− 0.16 [− 0.31; − 0.12]	0.604	**0.041**	
X5Hz, z-score	1.59 [0.58; 2.27]	1.48 [0.87; 1.67]	0.83 [0.67; 1.52]	0.88 [0.06; 1.72]	0.57 [0.16; 1.21]	0.76 [0.45; 2.11]			0.065
AX, kPa/L	1.34 [0.73; 2.76]	1.21 [0.68; 2.87]	0.39 [0.35; 2.97]	1.18 [0.50; 2.98]	1.16 [0.58; 2.06]	0.86 [0.41; 3.97]	0.328	**0.016**	
AX, % z-score	0.92 [0.70; 1.91]	1.25 [0.45; 1.73]	0.84 [0.31; 2.02]	0.82 [0.21; 2.75]	0.76 [0.00; 1.28]	0.90 [0.51; 2.63]			0.253
*eRIC model*									
R_central_RS, kPa/L/s	0.49 [0.32; 0.56]	0.44 [0.29; 0.52]	0.42 [0.29; 0.48]	0.49 [0.40; 0.60]	0.50 [0.40; 0.62]	0.41 [0.33; 0.63]	0.600	0.116	0.403
IRS × 100, kPa/L/s^2^	1.02 [0.90; 1.16]	0.91 [0.88; 1.01]	0.97 [0.90; 1.09]	0.97 [0.76; 1.15]	0.98 [0.75; 1.10]	0.95 [0.80; 1.13]	0.159	**0.023**	0.567
CpRS × 100, L/kPa	7.83 [5.57; 11.63]	7.15 [5.90; 12.48]	16.14 [5.51; 18.86]	8.80 [4.00; 15.28]	9.44 [7.00; 15.37]	11.05 [4.00; 16.34]	0.416	**0.011**	0.930
R_peripheral_RS, kPa/L/s	0.89 [0.68; 1.04]	0.71 [0.50; 0.94]	0.77 [0.62; 5.00]	1.00 [0.77; 1.67]	1.01 [0.66; 1.05]	0.67 [0.51; 1.14]	0.082	0.311	0.403

VD is the anatomical volume of airways; AD denotes Autogenic Drainage

^$^ The 7 children with airflow limitation who had a bronchodilator test had z-scores of FEV_1_ of − 2.72 [− 3.79; − 2.31] and FVC of − 1.55 [− 1.90; − 1.00] that have to be compared to the “After salbutamol” results

ND denotes not done. Bold *P* values are those < 0.05

The correlations between VD and height were highly significant (before physiotherapy: R = 0.91; after physiotherapy: R = 0.86, *p* < 0.0001 for both) justifying the normalisation

### Effect of chest physiotherapy (30 children)

**Fig. 3:**
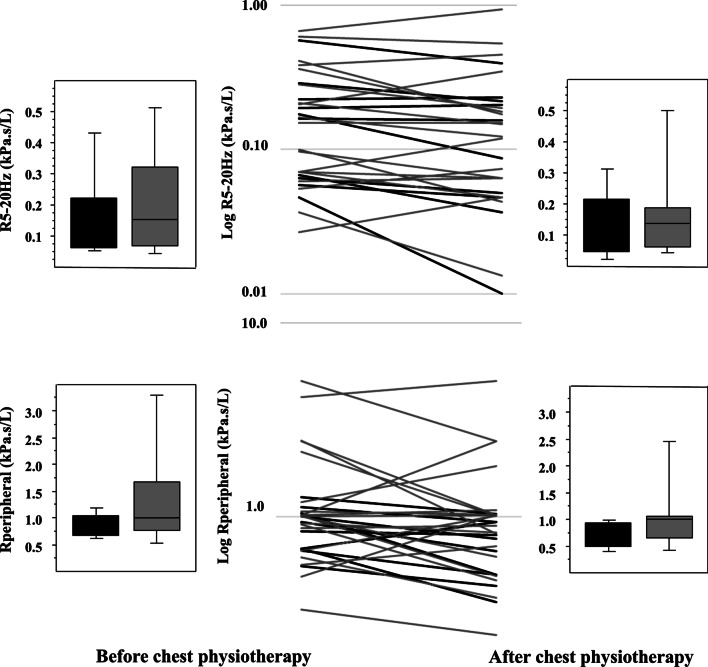
Indices of peripheral resistance assessed before and after AD. The upper panels describe R5-R20Hz resistance of the respiratory system before (left panel) and after (right panel) a single AD session. Box and whisker plots show median, 25th and 75th percentiles, and 10th and 90th percentiles in children with CF with (dark grey) and without (light grey) bronchial congestion. The middle panel describes individual data of the 30 children: black lines for the 10 children with bronchial congestion and light grey lines for the 20 children without bronchial congestion. To better highlight individual changes of R5–20 Hz, the Y-axis is a logarithmic base 10 scale. A significant decrease in the raw value of R5–20 Hz was observed in the children with bronchial congestion (*P* = 0.025) whereas it was not significant in those without congestion (*P* = 0.327). The lower panels describe R_peripheral_RS computed using eRIC model before (left panel) and after (right panel) a single AD session. Box and whisker plots show median, 25th and 75th percentiles, and 10th and 90th percentiles in children with CF with (dark grey) and without (light grey) bronchial congestion. The middle panel describes individual data of the 30 children: black lines for the 10 children with bronchial congestion and light grey lines for the 20 children without bronchial congestion. To better highlight individual changes of R_peripheral_RS, the Y-axis is a logarithmic base 10 scale. A significant decrease in R_peripheral_RS (*P* = 0.037) is evidenced after physiotherapy in the children with bronchial congestion, which was not significant in those without congestion (*P* = 0.390)

The effects of AD on two peripheral markers of resistance (R5–20 Hz and R_peripheral_RS) are described in Fig. 3, taken into account the presence or absence of bronchial congestion.

In the whole group (n = 30), R5–20 Hz significantly decreased after AD (*P* = 0.049, Table 1), which was related to a decrease in the 10 children with congestion (Fig. 3), whereas it was not significantly modified in the 20 children without congestion. The change in z-scores of R5–20 Hz (pre minus post AD value) in children with congestion was + 0.13 [− 0.03; + 0.79] and 0.00 [− 0.32; + 0.25] in those without congestion. R_peripheral_RS was not significantly modified after AD in the whole group. Nevertheless, it significantly decreased in the children with congestion (Fig. 3), whereas it was not modified in the no congestion group.

The other indices obtained from IOS or eRIC model were not significantly modified after AD (data not shown).

When the effects of AD were evaluated based on the opinion of the child (absence, n = 14 or presence of congestion, n = 16), similar results (a significant effect of AD in children with presence of congestion) were evidenced for both R5–20 Hz (*P* = 0.010) and R_peripheral_RS (*P* = 0.026).

AD did not significantly modify VD, neither in the whole population (*P* = 0.097) nor in children with (*P* = 0.138) or without (*P* = 0.489) bronchial congestion.

### Effect of salbutamol (14 children)

Bronchodilator administration was associated with a significant increase in FEV_1_ in the 14 children who performed postbronchodilator spirometry due to due to airflow limitation persisting after AD (Table 2), which was due to its effect in children having had bronchial congestion: *P* = 0.018 versus children without bronchial congestion (children without bronchial congestion, *P* = 0.172). Among the IOS indices, Fres (*P* = 0.016), AX (*P* = 0.016), R5Hz (*P* = 0.034) and X5Hz (*P* = 0.041) were significantly modified by the bronchodilator. The eRIC model showed significant modifications of IRS and CpRS (Table 2).

## Discussion

### Main results

The main result of our prospective study is to demonstrate that one session of AD performed by a physiotherapist has the ability to decrease two indices of peripheral resistance obtained from IOS measurement, specifically in children with CF with moderate to severe bronchial congestion. Additionally, bronchodilator administration after AD improved other indices of lung function, suggesting complementary benefits.

### Effect of physiotherapy on mucus clearance

Sputum weight was in agreement with the weight expected based on the literature on CF patients [21, 22]. The main effect of airway clearance techniques occurs in the presence of large volumes of sputum production in other bronchial diseases [1]. Our results in CF are similar, suggesting that the improvement in peripheral resistance of the respiratory system was related to mucus withdrawal from the distal airways in the children depicting moderate to severe bronchial congestion.

In a similar study, Wallaert et al. performed forced oscillation tests before and after a single AD session [17], but did not consider the degree of bronchial congestion. Their results showed a moderate decrease in inspiratory resistance in the global (Rrs_5_) and proximal (Rrs_11_ and Rrs_19_) airways, but not in the distal compartment (Rrs_5_–Rrs_19_) in 30 adult CF subjects [17]. The authors hypothesised that AD did not significantly increase the luminal calibre of the distal airways. They argued that the sputum coming from the proximal part of the lung is significantly cleared immediately after the AD session, in contrast to the more peripheral secretions. It must be emphasised that their adult population exhibited moderate to severe obstructive ventilatory disorders that may have prevented distal mucus clearance. Their results are in contradiction with those showing that cough only partially compensated for impaired mucociliary clearance and that airway clearance techniques were a necessary adjunct for the enhancement of impaired peripheral lung clearance [2].

### Effect of AD on resistance

The eRIC model adequately fitted the experimental data of children with CF, the modeling error was inferior to the error observed in asthmatic children [8]. It emphasizes that small airway indices (peripheral resistance and compliance of the respiratory system) are important contributors of respiratory system impedance in CF disease. We show that R5–20 Hz and peripheral resistance decreased by 30% and 20% (% baseline) respectively after AD in children with bronchial congestion, which is a small effect as expected. For instance, in the study of Pfleger and colleagues, the FEV_1_ significantly increased by 2.24% predicted or 80 mL after chest physiotherapy [23]. One may wonder whether this level of improvement is higher than the repeatability of IOS measurement and whether it is clinically significant. We show an excellent repeatability that was even better than that obtained by others [24]. Nevertheless, the change in z-score of R5–20 Hz is lower than its within-visit variability in children with bronchial congestion, which emphasizes that a significant statistical effect cannot be guaranteed in all participants. But, the level of R5–20 Hz decrease is within the range of values defining significant bronchodilator response (~ 15 to 40%) [24, 25]. This level of improvement is also in accordance with that observed following treatment of CF exacerbation [26–28].

Similarly, in a recent study, significant changes were observed in the values of R5–R20Hz and AX, after an oscillatory positive expiratory pressure session and thoracic compression, in the comparison with rest in adult patients with non-CF bronchiectasis, also suggesting peripheral effects of airway clearance techniques [29]. The beneficial effect of oscillatory positive expiratory pressure, combined with DNase, has also been recently shown in children/adolescents with CF who showed an immediate decrease in airway resistance and reactance [30].

### Effect of AD on anatomical dead space

AD, despite mucus clearance, was unable to increase the anatomical dead space in our study. This result could be related to the specific distal effect of AD that was evidenced using IOS measurements. Moreover, using functional respiratory imaging, Leemans and colleagues showed that the volume of the airways decreased following high-frequency chest wall oscillation in CF patients, whereas resistance increased [22]. They explained this result based on a mucus shift from the periphery towards the central lung regions [22], a proposition confirmed by fluid dynamics simulation works [31]. In this latter study, the authors showed that the main effect of chest physiotherapy is to reduce the hydrodynamic resistance of the airway tree by secretion redistribution in the tree [31].

### Effect of salbutamol

The long-term effects of bronchodilator administration in CF remain debated [32]. In the evaluation of bronchodilator response in 33 CF patients, Öztürk and colleagues found significant changes in IOS (decrease in AX, Fres and R5-R20Hz) and spirometry (increase in FEV_1_) after bronchodilator use [33]. We observed quite similar results. It has been shown that sympathomimetics relieve bronchospasm in many CF subjects, but since they enhance airway compressibility, they thereby decrease peripheral expiratory airflow in some subjects [34]. The increase in respiratory system compliance that is demonstrated using the eRIC model is in agreement with the enhancement of airway compressibility. Overall, the effects of bronchodilator administration seem complementary to those of AD.

### Study limitations

Our study has limitations that are related to its design. Only short-term effects of AD were evaluated in a restricted sample of children with CF. The two-group subdivision may seem artifactual since sputum production during the AD session was a continuum, but similar results were obtained based on the child’s opinion. Moreover, in the whole group, a decrease in R5–R20Hz was demonstrated. Our results were obtained in children with mildly affected lung function and may not be generalizable to all subjects with CF. The predicted values of IOS that have been used were developed in Mexican children [13], which may constitute a limitation; but their adequacy for Caucasian children has further been demonstrated [35].

Sputum elimination was also determined in the present study. For this purpose, the wet sputum weight was measured immediately after the interventions. It is well known that mucus quantity, as volume or weight, cannot represent mucus depuration without considering the possible losses by swallowing or errors in its collection [36]. Furthermore, saliva contamination can represent a confounding factor for this measurement. Even if the assessment of bronchial congestion using auscultation and sputum weight has inherent limitations, their correlation is reassuring; the strong correlation (r value 0.6 to 0.8) evidenced also suggests that interobserver variability of bronchial congestion assessment was restricted, may be due to the involvement of experienced and specialized physiotherapists, which is a limitation for the generazibility of our results since different physiotherapists could obtain different results, even using a similar technique. Computer Aided Lung Sound Analysis is proposed as an objective, non-invasive, bedside clinical measure with the potential to monitor and assess the effects of airway clearance therapy [37], which remains to be demonstrated. Whether other kinds of airway clearance techniques can afford similar benefits remains to be evaluated and it remains to be highlighted that AD in our study improved lung function in children with bronchial congestion. Finally, since our study was not a randomized clinical trial, we show that AD is associated with a change in R5–20 Hz but a causal relationship cannot be formally inferred. The changes evidenced were very small and probably not associated with any short-term clinical effect. This was beyond the subject of our study, which focused on the distal effects of AD, which deserved to be demonstrated.

In conclusion, one session of AD performed by a physiotherapist has the ability to decrease peripheral resistance obtained from IOS measurements, more specifically in children with CF with moderate to severe bronchial congestion.

## Data Availability

The datasets used and/or analysed during the current study are available from the corresponding author on reasonable request.
